# The Hospital World

**Published:** 1910-05-28

**Authors:** 


### The Hospital World.

### Personalia.

Owing to illness Mr. John Wainwright, who has acted on the Oakwell Joint Hospital Board for a period long enough to make him a well-known figure, has resigned his post. This is particularly to be regretted because Mr. Wainwright's connection with the Hospital Board has been conterminous with the Board's existence. It is the people of Birstall, however, with whom this regret will be the liveliest, and it is they who will endorse particularly the vote of condolence, and the expression of the Board's hopes of Mr. Wainwright's return, which was passed at the recent annual meeting.

We regret to announce the death, after a few days' illness, of Mr. J. S. Barber, who recently resigned the post of senior dispenser to the Royal Free Hospital, which he had held for the remarkably long period of fifty-nine years. On the occasion of his retirement from active work the committee of the hospital granted Mr. Barber a substantial pension in recognition of his long and faithful service, and the members of the committee, medical and nursing staff also presented him with testimonials as tokens of their esteem. All connected with the Royal Free Hospital much regret that Mr. Barber has not been spared to enjoy longer his well-earned rest from active work.

Two old members of the St. Mary's Hospital board of management, Col. W. C. Phillpotts and Mr. M. P. Christie, died last year, and their record of work indicates that they regarded their positions as a loving trust and a generous responsibility. Colonel Phillpotts had sat for seven years, and Mr. Christie for ten. Colonel Phillpotts brought to his duties the qualities to be expected from an officer of repute, who did not forget the traditions of the service in his civic duties, and Mr. Christie extended his activities to the generous point of being a well-known figure as a hospital visitor, whom patients and the nurses too will miss sadly. Moreover, his purse was as ready as his energy, and on both grounds St. Mary's has had to thank him again and again for his quick response to her necessities.

Among the resignations that St. Mary's Hospital has to record are those of Mr. James R. Mellor, who for eighteen years has been chairman of the board of management. The constant service which these years have served to mark, and for which the hospital is happy in remembering him, have made his election to a vice-presidency not only a gracious but also a well-deserved honour, which the hospital is never likely to regret. Another resignation which St. Mary's cannot well afford is that of Mr. H. A. Harben, chairman for six years of the board of management. No passage in the hospital's report is happier than the one that thanks him, which we quote here: "The board accepted his resignation with regret, and if it is now silent as to the important services rendered by Mr. Harben to the institution, it is only because it has the good fortune still to number him, as vice-president and treasurer, among its members."

### Elections and Appointments.

Mr. W. Austen Leigh has been elected deputy chairman of St. Mary's Hospital, Paddington.

Mr. A. Jones has been re-elected chairman of the Ormskirk and Lathom and Burscough Joint Hospital Board.

Mr. F. Ashe has taken the place of Mr. Willis on the Management Committee of the Swindon and District Hospital Board.

Sir William Watson, D.L., has been elected a vice-president of the Rotunda Hospital, Dublin, at a recent charter meeting of the governors.

Mr. H. A. Harben has resigned the chairmanship of the Board of Management of St. Mary's Hospital, of which he remains vice-president and treasurer.

Mr. Alderman Ben Turner has been elected chairman, and Mr. J. Garbutt has been elected vice-chairman of the Oakwell Joint Hospital Board at the annual meeting held this month (May).

At the quarterly meeting of the Governors of the Forres Leanchoil Hospital, the following were appointed to form the House Committee for the ensuing six months: Rev. James Hendry (June), Mr. James Gray (July), Mr. W. A. Robertson (August), Mr. W. G. Ross (September), Mr. Patrick Butler (October), and Provost Douglas (November).

Colonel Bannerman, Mr. Henry Gardener, and Mr. E. Parker Young have been re-elected ordinary members of the Board of Management of St. Mary's Hospital. Sir R. Melvill Beachcroft, Mr. H. H. Fuller, Mr. A. R. Prideaux, and Mr. H. E. Verey, appointed during the year to fill vacancies, have been re-elected also.

The Rev. W. Baillie, M.A.; Viscount Bangor, Mr. W. F. Bewley, Mr. Robert Booth, J.P.; the Rev. W. Crawford, the Rev. Charles Dowse, Mr. Hamilton Drummond, J.P.; Mr. Edwin Hamilton, J.P.; Mr. Charles Leeper, Mr. John Leech, K.C.; the Rev. Canon Mahaffy, Mr. John Parkes, the Rev. S. Prenter, D.D.; Mr. A. D. Price, Captain Riall, D.L.; Mr. J. W. Richards, the Rev. E. Robinson, the Rev. John Stewart, Mr. Edmund Trouton, and Mr. D. A. Maxwell have been elected to form the Managing Committee of the Adelaide Hospital, Dublin. The Hon. Secretaries are Captain R. Wade Thompson, J.P., and Dr. Wallace Beatty, M.D.; the Hon. Treasurer is Mr. David Ross, J.P.

The committee for the present year at the Bath Royal Mineral Hospital will include the Mayor of Bath, the Rev. C. E. B. Barnwell, Dr. Bannatyne, Dr. Carter, Col. Tredway Clarke, Sir Robert Cockburn, Dr. A. E. W. Fox, Dr. Henry Gervis, Dr. Preston King, Col. Kirkwood, the Rev. G. F. Pearson, Major J. M. T. Reilly, Col. Ricketts, Lieut.-Col. J. H. Seagram, Col. Skrine, Sir John Stevens, Major Waterhouse, the Rev. H. H. Winwood, Mr. J. E. Goddard Bradford, Mr. W. S. Brymer, Mr. R. C. Bush, Mr. J. Russell Cox, Mr. Russell Duckworth, Mr. T. M. Gibson, Mr. W. Handyside, Mr. J. Holst, Mr. F. W. Lawrence, Mr. G. H. B. Moger, Mr. J. A. Nunneley, Mr. E. E. Phillips, Mr. H. W. B. Schofield, and Mr. A. H. Unwin.

